# Prevalence of oral mucosal alterations in type 2 diabetes mellitus patients attending a diabetic center

**Published:** 2014

**Authors:** Syed Fareed Mohsin, Syed Azhar Ahmed, Asher Fawwad, Abdul Basit

**Affiliations:** 1 Syed Fareed Mohsin, BDS, MSc (London), MFD RCS(Ireland), MFDS RCPSG, PhD, Department of Oral Medicine/Oral Pathology, Baqai Dental College, Karachi, Pakistan.; 2 Syed Azhar Ahmed, MBBS, PhD (London),FRC, Path.(London), FCPS (Pak), Director Post graduate Education, Baqai Medical University Karachi, Pakistan.; 3 Asher Fawwad, MBBS, MPhil, Department of Biochemistry, Baqai Medical University, Karachi, Pakistan.; 4 Abdul Basit, MBBS, FRCP, Director Baqai institute of Diabetology and Endocrinilogy, Karachi, Pakistan.

**Keywords:** Oral mucosal lesions, Potentially malignant disorders, Type 2 diabetes mellitus

## Abstract

***Objective:*** To explore an association between oral mucosal alterations and type 2 Diabetes mellitus.

***Methods: ***This study was conducted at Baqai Institute of Diabetology and Endocrinology and Baqai Medical University from September 2010 to September 2012. A total of 800 individuals’ 395 type 2 diabetes mellitus patients and 405 healthy individuals were enrolled in this study. An oral clinical examination was carried out for all participants using a mouth mirror, visible light source and cotton gauze.

***Results: ***The prevalence of oral mucosal lesions was high significantly < 0.0001; odd ratio 2.601, CI 1.929-3.509 in type 2 diabetic as compared to non-diabetic. With respect to specific oral mucosal lesions, highly significant association p<0.0001; Odd ratio 4.275, CI 2.798-6.534 was found between coated tongue with type 2 diabetes mellitus. This study did not find any association (p>0.05) between type 2 diabetes mellitus and potentially malignant disorders.

***Conclusion: ***This study showed that the prevalence of oral mucosal lesions was higher in type 2 diabetic than non-diabetics. This study provides evidence that diabetes has a negative influence on oral health.

## INTRODUCTION

Oral diseases have a direct adverse effect on quality of life and may have serious impact on individual’s well being, self esteem, financial status and social interaction^1^. Although oral diseases are often not life threatening conditions, poor oral health results in diminished overall health whereas optimum oral health improves quality of life.^2^

Diabetes mellitus is recognized as a group of metabolic disorders characterized by chronic hyperglycemia and glucose intolerance, due to insulin deficiency, impaired effectiveness of insulin action or both.^3^ Diabetes mellitus (DM) is a major public health concern worldwide. The number of diabetic individuals is increasing because of population growth, aging, increasing prevalence of obesity and urbanization^4^. It has been reported that the number of DM individuals worldwide will rise from 171 million (in 2000) to 366 million by 2030.^5^

The prevalence of DM in Pakistan is 12% in people over 25 years of age, which is not surprising considering the presence of associated risk factors for DM in Pakistan. The number of DM individuals in Pakistan was 5.2 million in year 2000, with a predicted increase to 13.9 million by the year 2030.^5^

Diabetes frequently predisposes an individual to oral health complications. These complications include multiple oral soft tissue pathologies and inflammatory diseases.^6^ Studies have shown that diabetes mellitus may cause diseases like periodontitis, gingivitis, stomatitis, angular chelitus and various tongue disorders.^7^^,^^8^ Diabetes leads an individual to bacterial and fungal oral infections.^9^

In comparison with studies on dental caries and periodontal diseases epidemiological studies on oral mucosal lesions are rare globally.^10^ Some studies have reported a high prevalence of oral mucosal lesions in DM patients.^11^^,^^12^ However, it has not been proven whether oral mucosal lesions in DM patients occur more frequently than in those not suffering from DM.^13^^,^^14^

The aim of this study was to determine the association between oral mucosal alterations and type 2 diabetes mellitus.

## METHODS

This study was approved by ethical review committee of Baqai Medical University. The study was conducted at Baqai Institute of Diabetology and Endocrinology (BIDE) and Baqai Medical University (BMU). Previously diagnosed type 2 DM patients were recruited from BIDE and healthy individuals from BMU. 

The present cross-sectional study was carried out in 395 type 2 diabetes mellitus patients and 405 healthy individuals. All the participants (with and without Type 2 Diabetes) underwent a systematic clinical examination of the oral mucosa. The oral examination was carried out using visible light, a dental mirror and cotton gauze. Biopsies were also carried out to confirm the diagnosis if required. The oral mucosal alterations were classified in two types; Benign Oral Mucosal Lesions (BML) and Potentially Malignant Disorders (PMDs). Benign oral mucosal lesions included a diagnosis of coated tongue, fissured tongue, geographic tongue, melanin pigmentation, linea alba and fordyces granules. Potentially malignant disorders includes in this study were lichen planus, leukoplakia and oral submucous fibrosis, while mixed lesions comprised of benign oral mucosal lesions and potentially malignant disorder.

***Inclusion criteria:***

- Previously diagnosed under treatment Type 2 diabetics with no other complications. Patients were assessed and described by physician as having no major micro/macro vascular complications related to diabetes mellitus.
- Non-diabetic individuals accessed by fasting blood glucose level without other illness and similar socio-economic level and age were recruited as healthy subjects.

***Exclusion criteria:***

- Immunologically related disorders to be associated with lichen planus like ulcerative colitis, chronic active hepatitis, myasthenia gravis.
- Patients with type 1 Diabetes.

***Sample Size Calculation: ***Sample size was calculated by using WHO sample size calculator (sample size determination in health studies, a practical manual, software version by KC Lun and Peter Chiam, National University of Singapore).Choosing confidence level of 95%, anticipated proportion of oral mucosal lesions in type 2 diabetes Mellitus as 50% and power of the study as 80%.

***Statistical Analysis: ***SPSS (version 19) was used for data entry and analysis. The association between diabetes and oral mucosal lesions was determined by using chi-square test. Significant differences were recorded at p<0.05.

## RESULTS

***Demographic Profile:*** A total of 800 individuals; 482 (60.3%) male and 318 (39.7%) female participants underwent clinical oral examination. The mean age for male and female participants was 51(± 8.85) years and 49 (± 8.9) years respectively. Among 395 DM type 2 patients, 53.7% were male and 46.3% female; with the mean age of male 53 (±9.8) years and female 53(±8.8) years. In 405 healthy individuals; male to female ratio was 66.7% and 33.3% respectively, with mean age of male 48(±7.2) years and female 44(±5.8) years.

***Oral Mucosal Lesions in Diabetics and Non-Diabetics:*** The prevalence of oral mucosal lesions was significantly high in type 2 diabetes mellitus patients (p<0.0001) as compared with healthy non-diabetic individuals (Table-I).

**Table-I:** Oral mucosal lesions in diabetics and non-diabetics

***Oral Mucosal Lesions***	***Type 2 diabetics*** *n(%)*	***Non-diabetics*** *n (%)*	***Odd ratio***	***Confidence interval***	***P value***
Benign Oral Mucosal Lesions(BML)	199 (50.4)	117 (28.9)	2.601	1.929-3.509	<0.0001
Potentially Malignant Disorder(PMD)	13 (3.3)	20 (4.9)	0.994	0.482-2.052	0.987
Mixed Lesions (BML)+(PMD)	13 (3.3)	08 (2.0)	2.485	1.009-6.123	0.048

***Distribution of Oral Lesions:***

**Table-II:** Distribution of oral lesions in diabetics and non-diabetics

***Oral Mucosal Lesions(OML)***	***Variables***	***Type 2 Diabetics*** *n(%)*	***Non-Diabetics*** *n(%)*	***Odd ratio***	***95% Confidence interval***	***p-value***
Oral Mucosal Lesions(OML)	Subject with one or more lesions(BML+PMD)	225 (60.8)	145 (39.2)	2.530	1.901-3.366	<0.0001
	Coated Tongue	106 (26.8)	32 (7.9)	4.275	2.798-6.534	<0.0001
	Fissured Tongue	63 (15.9)	40 (9.9)	1.732	1.134-2.644	0.011
	Melanin Pigmentation	60 (15.2)	45 (11.1)	1.433	0.947-2.168	0.089
	Linea Alba	31 (7.1)	12 (3.0)	2.789	1.411-5.513	0.003
	Fordyces Granules	9(2.3)	0(0.0)			
	Geographic Tongue	5 (1.3)	4(1.0)	1.285	0.343-4.822	0.710
	Total	274 (68.6)	133 (32.9)			
Potentially Malignant Disorders(PMD)	Leukoplakia	14 (3.5)	12 (3.0)	1.203	0.550-2.635	0.643
	Lichen Planus	7(1.8)	4(1.0)	1.809	0.525-6.227	0.348
	Oral Submucous Fibrosis	8(2.0)	12 (3.0)	0.677	0.274-1.674	0.399

## DISCUSSION

An association of diabetes as a risk factor for oral diseases has been extensively discussed in several studies.^15^^,^^16^ In this study, prevalence of oral mucosal lesions was significantly high in type 2 diabetics as compared to non-diabetics. With respect to specific oral mucosal lesions a highly significant association was observed between coated tongue and type 2 diabetes. This finding can be related to decrease in salivary flow and a high salivary viscosity that result in reduced action of salivary antimicrobials factors and cleaning capacity of the tongue. A study carried out on 146 type 2 DM patients also found a prevalence of 28.7% of coated tongue.^17^

An association between diabetes and fissured tongue has been reported in a previous study.^18^ In the present study this alteration was more common in diabetic patients than in the non-diabetics. Another study also showed a similar (17.8%) prevalence of fissured tongue among type 2 diabetics.^17^

The present study did not find any association between DM type 2 and potentially malignant disorder. An association has been reported between diabetes mellitus and premalignant oral lesions among Keralite women in India.^18^ Another study also reported a significantly higher prevalence of potentially malignant disorder including leukoplakia among type 2 diabetes mellitus patients when compared to non-diabetics.^17^ The prevalence of leukoplakia in the present study was only 3.5% in type 2 diabetic individuals with no significant difference as compared with the non-diabetics. The high prevalence of leukoplakia in above mentioned study could be attributed to a high number of smoker’s among the diabetics. The DM type 2 patients who smoke are more prone to leukoplakia as compared to those who do not smoke.^19^ The present study had less number of smokers compared with the other similar studies.^17^

The association between oral lichen planus and diabetes mellitus was first described in 1966.^20^ Many authors have reported a strong association between lichen planus and diabetes mellitus.^21^^,^^22^ In the present study, lichen planus was diagnosed in 1.8% of type 2 diabetes mellitus patients, with no significant difference from the non-diabetic group. Similar to our result, another study also found a low prevalence of lichen planus (0.55%) in diabetic patients.^23^ Some authors have suggested that oral lichen planus in diabetes mellitus patients could be linked with compromised immune system in these patients.^24^ Lichenoid lesions among diabetic patients may be related to a number of oral hypoglycemic medications taken particularly by older individuals.^25^

Oral premalignant and malignant conditions are highly prevalent in South East Asia. The South District of Karachi has the highest incidence of oral cancers in the world.^26^ Few such conditions were found in this study, probably because people may seek help for mucosal lesions in several other institutions.

## CONCLUSION

The prevalence of benign oral mucosal lesions was significantly high < 0.0001 in type 2 DM patients than the non-diabetics in the study sample. No significant (p>0.05) association was found between Type 2 Diabetes Mellitus and potentially malignant oral mucosal lesions.

## Authors Contribution:

**SFM: **Did data collection and manuscript writing.

**AF and AB: **Did statistical analysis & editing of manuscript.

**SAA: **Did review and final approval of manuscript.
